# Central venous-to-arterial carbon dioxide difference combined with arterial-to-venous oxygen content difference is associated with lactate evolution in the hemodynamic resuscitation process in early septic shock

**DOI:** 10.1186/s13054-015-0858-0

**Published:** 2015-03-28

**Authors:** Jaume Mesquida, Paula Saludes, Guillem Gruartmoner, Cristina Espinal, Eva Torrents, Francisco Baigorri, Antonio Artigas

**Affiliations:** Critical Care Center, Hospital de Sabadell, Corporació Sanitària Universitària Parc Taulí, Universitat Autònoma de Barcelona, Parc Tauli, 1, Sabadell, 08208 Spain

## Abstract

**Introduction:**

Since normal or high central venous oxygen saturation (ScvO_2_) values cannot discriminate if tissue perfusion is adequate, integrating other markers of tissue hypoxia, such as central venous-to-arterial carbon dioxide difference (P_cva_CO_2_ gap) has been proposed. In the present study, we aimed to evaluate the ability of the P_cva_CO_2_ gap and the P_cva_CO_2_/arterial-venous oxygen content difference ratio (P_cva_CO_2_/C_av_O_2_) to predict lactate evolution in septic shock.

**Methods:**

Observational study. Septic shock patients within the first 24 hours of ICU admission. After restoration of mean arterial pressure, and central venous oxygen saturation, the P_cva_CO_2_ gap and the P_cva_CO_2_/C_av_O_2_ ratio were calculated. Consecutive arterial and central venous blood samples were obtained for each patient within 24 hours. Lactate improvement was defined as the decrease ≥ 10% of the previous lactate value.

**Results:**

Thirty-five septic shock patients were studied. At inclusion, the P_cva_CO_2_ gap was 5.6 ± 2.1 mmHg, and the P_cva_CO_2_/C_av_O_2_ ratio was 1.6 ± 0.7 mmHg · dL/mL O_2_. Those patients whose lactate values did not decrease had higher P_cva_CO_2_/C_av_O_2_ ratio values at inclusion (1.8 ± 0.8vs. 1.4 ± 0.5, *p* 0.02). During the follow-up, 97 paired blood samples were obtained. No-improvement in lactate values was associated to higher P_cva_CO_2_/C_av_O_2_ ratio values in the previous control. The ROC analysis showed an AUC 0.82 (*p* < 0.001), and a P_cva_CO_2_/C_av_O_2_ ratio cut-off value of 1.4 mmHg · dL/mL O_2_ showed sensitivity 0.80 and specificity 0.75 for lactate improvement prediction. The odds ratio of an adequate lactate clearance was 0.10 (*p* < 0.001) in those patients with an elevated P_cva_CO_2_/C_av_O_2_ ratio (≥1.4).

**Conclusion:**

In a population of septic shock patients with normalized MAP and S_cv_O_2_, the presence of elevated P_cva_CO_2_/C_av_O_2_ ratio significantly reduced the odds of adequate lactate clearance during the following hours.

## Introduction

Current guidelines for hemodynamic management of severe sepsis and septic shock recommend the use of global markers of tissue hypoxia as resuscitation endpoints [1,2]. In the initial resuscitation period, targeting either central venous oxygen saturation (S_cv_O_2_) normalization or lactate clearance, or the combination of both, is accepted [1,3,4]. However, each one of these two variables has their own limitations. Although the use of S_cv_O_2_ seems to provide more real-time information than lactate clearance, the nature of septic conditions, characterized by microcirculatory heterogeneity that generates capillary shunting, is frequently accompanied by elevated S_cv_O_2_ values. Indeed, abnormally high S_cv_O_2_ values have been associated with increased mortality in septic shock patients [5,6]. Achievement of the recommended normalized S_cv_O_2_ values during the initial resuscitation therefore does not rule out persistent tissue hypoxia, and some authors consider that S_cv_O_2_ should be used in combination with other tissue perfusion endpoints [7]. On the other hand, despite lactate clearance being proven to be as beneficial as S_cv_O_2_ in guiding resuscitation in sepsis, at the bedside the clinician has to face the uncertainty of a high lactate value, without knowing whether this lactate reflects persistence of hypoperfusion or whether its normalization is just a matter of time [8]. On the whole, elevated lactate values could lead to unnecessary interventions, with their potential deleterious effects, such as tissue edema and increased fluid balance, which have consistently been associated with worse outcome [9].

Recently, some authors have advocated that the mixed and/or central venous-to-arterial carbon dioxide difference (P_cva_CO_2_ gap) might be complementary tools to identify patients with persistent global hypoperfusion [10]. Certainly, both partial pressure of carbon dioxide gaps have demonstrated their prognostic value in different conditions [11-14], and a cutoff value of 6 mmHg seems to reflect whether global flow is adequate (gap >6 mmHg) or insufficient (gap ≥6 mmHg). In addition, some authors have suggested that correcting the P_cva_CO_2_ gap by an approximation of the oxygen consumption, the P_cva_CO_2_/arterial-to-venous oxygen content difference (C_av_O_2_) ratio, might be superior to the P_cva_CO_2_ gap to detect anaerobic metabolism [15], and therefore should be a more reliable parameter to guide the resuscitation process.

In the present study, we addressed the question of whether the P_cva_CO_2_ gap and the P_cva_CO_2_/C_av_O_2_ ratio are useful in predicting the evolution of lactate, reflecting the persistence of tissue hypoperfusion and/or anaerobic metabolism. For that purpose, we studied a well-defined population of patients with severe sepsis or septic shock, once normalized S_cv_O_2_ values were achieved.

Preliminary data from this study were presented at the 27th European Society of Intensive Care Medicine (ESICM) Annual Congress.

## Methods

### Setting

We conducted a retrospective observational study in a 30-bed mixed ICU at a university hospital. Study approval and waived consent were authorized by the local ethics committee (Comitè Ètic d’Investigació Clínica, Fundació Parc Taulí; Reference CEIC 2014637).

### Patients and data collection

Septic shock patients within the first 24 hours of ICU admission were studied. Severe sepsis and septic shock were defined according to international sepsis definitions [16]. All patients were resuscitated following the Surviving Sepsis Campaign guidelines [1]. Exclusion criteria were: age under 18 years, and the presence of an uncontrolled source of infection. Once normalized values of mean arterial pressure (MAP ≥65 mmHg) and S_cv_O_2_ (≥70%) were achieved, and the medical team in charge decided not to perform further resuscitation interventions (such as volume expansion and/or changes in inotropic or vasopressor drugs), simultaneous blood samples were obtained from a central venous line and an arterial catheter. The investigators confirmed the correct positioning of the venous catheter tip on chest X-ray examinations. Measured variables included arterial oxygen tension (P_a_O_2_), arterial carbon dioxide tension (P_a_CO_2_), central venous oxygen tension (P_cv_O_2_), and central venous carbon dioxide tension (P_cv_CO_2_). Arterial oxygen saturation (S_a_O_2_) and S_cv_O_2_ were calculated from the oxyhemoglobin dissociation curve. Arterial and central venous lactate, and hemoglobin concentration (Hb) were also measured. The arterial oxygen content (C_a_O_2_), central venous oxygen content (C_v_O_2_), C_av_O_2_, oxygen extraction ratio (O_2_ER), P_cva_CO_2_ gap, and P_cva_CO_2_/C_av_O_2_ ratio were calculated according to the following formulas:

$$ \cdotp {\mathrm{C}}_{\mathrm{a}}{\mathrm{O}}_2=\left(1.34\times {\mathrm{S}}_{\mathrm{a}}{\mathrm{O}}_2\times \mathrm{H}\mathrm{b}\right)+\left(0.003\times {\mathrm{P}}_{\mathrm{a}}{\mathrm{O}}_2\right) $$

$$ \cdotp {\mathrm{C}}_{\mathrm{cv}}{\mathrm{O}}_2=\left(1.34\times {\mathrm{S}}_{\mathrm{cv}}{\mathrm{O}}_2\times \mathrm{H}\mathrm{b}\right)+\left(0.003\times {\mathrm{P}}_{\mathrm{cv}}{\mathrm{O}}_2\right) $$

$$ \cdotp {\mathrm{C}}_{\left(\mathrm{a}-\mathrm{v}\right)}{\mathrm{O}}_2={\mathrm{C}}_{\mathrm{a}}{\mathrm{O}}_2-{\mathrm{C}}_{\mathrm{cv}}{\mathrm{O}}_2 $$

$$ \cdotp {\mathrm{P}}_{\mathrm{cv}\mathrm{a}}{\mathrm{CO}}_2\mathrm{gap}={\mathrm{P}}_{\mathrm{cv}}{\mathrm{CO}}_2-{\mathrm{P}}_{\mathrm{a}}{\mathrm{CO}}_2 $$

$$ \cdotp {\mathrm{P}}_{\mathrm{cva}}{\mathrm{C}\mathrm{O}}_2/{\mathrm{C}}_{\mathrm{av}}{\mathrm{O}}_2\mathrm{ratio}={\mathrm{P}}_{\mathrm{cva}}{\mathrm{C}\mathrm{O}}_2\mathrm{gap}/{\mathrm{C}}_{\left(\mathrm{a}-\mathrm{v}\right)}{\mathrm{O}}_2 $$

$$ \cdotp {\mathrm{O}}_2\mathrm{E}\mathrm{R}={\mathrm{C}}_{\mathrm{a}\mathrm{v}}{\mathrm{O}}_2/{\mathrm{C}}_{\mathrm{a}}{\mathrm{O}}_2 $$

All consecutive paired blood samples (arterial and central venous) obtained for each studied patient within the following 24 hours, as indicated by the medical team, were also collected.

Patient demographics, diagnosis at ICU admission, sepsis origin, and Simplified Acute Physiology Score II score were recorded at inclusion. Hemodynamic variables (heart rate and arterial pressure) were recorded by routine bedside monitoring (Monitor Intellivue MP 70; Phillips Medizinsystems, Boeblingen, Germany). Arterial and central venous blood gas samples were analyzed using point-of-care equipment (ABL 700 series; Radiometer Medical, Copenhagen, Denmark).

Patients were followed during the whole ICU stay, and the ICU length of stay and ICU mortality were computed.

### Statistical analysis

Statistical analysis was performed by means of IBM SPSS statistics 20.0 software (IBM Corporation, Armonk, New York, USA). Normal distribution of the studied variables was confirmed using the Kolmogorov–Smirnov test. Accordingly, continuous variables were expressed as mean ± standard deviation, and categorical variables were expressed as the absolute number and proportions (%). A descriptive analysis was performed. Correlations between P_cva_CO_2_ gap, P_cva_CO_2_/C_av_O_2_ ratio, S_cv_O_2_, and lactate were explored using the Pearson test. According to previous published studies exploring the value of lactate clearance, lactate improvement was defined as the decrease of at least 10% of the previous lactate value [3,17]. Comparisons between lactate improvers and nonimprovers were performed with the Mann–Whitney *U* test (first set of measurements) and Student’s *t* test (for the whole paired measurements) for continuous variables, and with the chi-square test or Fisher’s exact test for categorical variables. The ability of the P_cva_CO_2_/C_av_O_2_ ratio for predicting a decrease in lactate ≥10% was calculated using a receiver operator characteristic (ROC) curve, and a clinically relevant cutoff value was computed. Binary logistic regression was used to determine the odds ratio of an adequate lactate clearance for the P_cva_CO_2_ gap and the P_cva_CO_2_/C_av_O_2_ ratio. Two-tailed *P* <0.05 was taken to indicate statistical significance.

## Results

Thirty-five septic shock patients were studied. Demographic, hemodynamic, and metabolic characteristics are presented in Table 1. At inclusion, patients had values of MAP 78 ± 12 mmHg, S_cv_O_2_ 71 ± 8%, and venous lactate 39 ± 49 mg/dl. The calculated P_cva_CO_2_ gap was 5.6 ± 2.1 mmHg, and the P_cva_CO_2_/C_av_O_2_ ratio was 1.6 ± 0.7 mmHg · dl/ml O_2_. No correlation between simultaneous S_cv_O_2_ and lactate was observed. The P_cva_CO_2_ gap at inclusion was inversely correlated to S_cv_O_2_ (*r* = –0.7, *P* <0.001), whereas the P_cva_CO_2_/C_av_O_2_ ratio directly correlated to lactate values (*r* = 0.73, *P* <0.001). The first consecutive paired blood samples were obtained after 3 ± 2 hours, none of them following additional resuscitation interventions. When compared with patients whose lactate values decreased, those patients whose lactate values did not decrease showed similar baseline P_cva_CO_2_ gap values at inclusion (6.1 ± 2.3 vs. 5.1 ± 1.9 mmHg, *P* = 0.09), whereas significantly higher P_cva_CO_2_/C_av_O_2_ ratio values at inclusion were observed (1.8 ± 0.8 vs. 1.3 ± 0.4 mmHg · dl/ml O_2_, *P* = 0.02). No differences in initial S_cv_O_2_ or lactate were observed according to lactate clearance (Table 1). The P_cva_CO_2_/C_av_O_2_ ratio ROC analysis showed an area under the curve of 0.75 (95% confidence interval = 0.6 to 0.92, *P* = 0.01) for adequate initial lactate clearance prediction.

**Table 1:** **Patient demographic, hemodynamic, and metabolic characteristics at inclusion**

	**All** **(** ***n*** **= 35)**	**Lactate improvers ** **(** ***n*** **= 17)**	**Lactate nonimprovers ** **(** ***n*** **= 18)**	***P*** **value**
Age (years)	65 ± 13	63 ± 14	69 ± 11	0.2
Male	22 (63)	10 (59)	12 (67)	0.8
Etiology				
Respiratory	8 (23)	4 (24)	4 (22)	
Abdominal	14 (40)	7 (41)	7 (39)	0.8
Urinary tract	4 (11)	3 (18)	1 (6)	
Soft tissue	5 (14)	1 (6)	4 (22)	
Other	4 (11)	2 (12)	2 (11)	
SAPS II	49 ± 11	47 ± 9	50 ± 12	0.5
SOFA score (day 1)	9 ± 3	9 ± 3	9 ± 3	0.6
Mechanical ventilation	28 (80)	14 (82)	14 (78)	0.5
Heart rate (beats per minute)	103 ± 14	103 ± 17	104 ± 13	0.8
MAP (mmHg)	78 ± 12	82 ± 11	71 ± 10	0.08
Norepinephrine use (%)	100	100	100	1
Norepinephrine dose (μg/kg/minute)	0.86 ± 0.65	0.66 ± 0.5	1.01 ± 0.75	0.05
Hemoglobin (g/dl)	11.2 ± 2.0	12.2 ± 1.7	9.9 ± 2.0	0.02
S_cv_O_2_ (%)	71 ± 8	71 ± 8	72 ± 8	0.7
Lactate (mg/dl)	38 ± 48	30 ± 15	46 ± 65	0.8
P_cva_CO_2_ gap (mmHg)	5.6 ± 2.1	5.1 ± 1.9	6.1 ± 2.3	0.09
P_cva_CO_2_/C_av_O_2_ ratio (mmHg · dl/ml O_2_)	1.6 ± 0.7	1.3 ± 0.4	1.8 ± 0.8	0.02
O_2_ER	0.26 ± 0.09	0.25 ± 0.09	0.25 ± 0.08	0.9
ICU length of stay (days)	15 ± 10	17 ± 14	13 ± 10	0.5
Mortality	10 (29)	3 (18)	7 (39)	0.2

Data presented as mean ± standard deviation or number (%). MAP, mean arterial pressure; O_2_ER, oxygen extraction ratio; P_cva_CO_2_ gap, central venous-to-arterial carbon dioxide difference; P_cva_CO_2_/C_av_O_2_ ratio, central venous-to-arterial carbon dioxide difference/arterial-to-central venous oxygen content difference ratio; SAPS, Simplified Acute Physiological Score; ScvO_2_, central venous oxygen saturation; SOFA, Sequential Organ Failure Assessment.

### Twenty-four-hour follow-up

**Figure 1:**
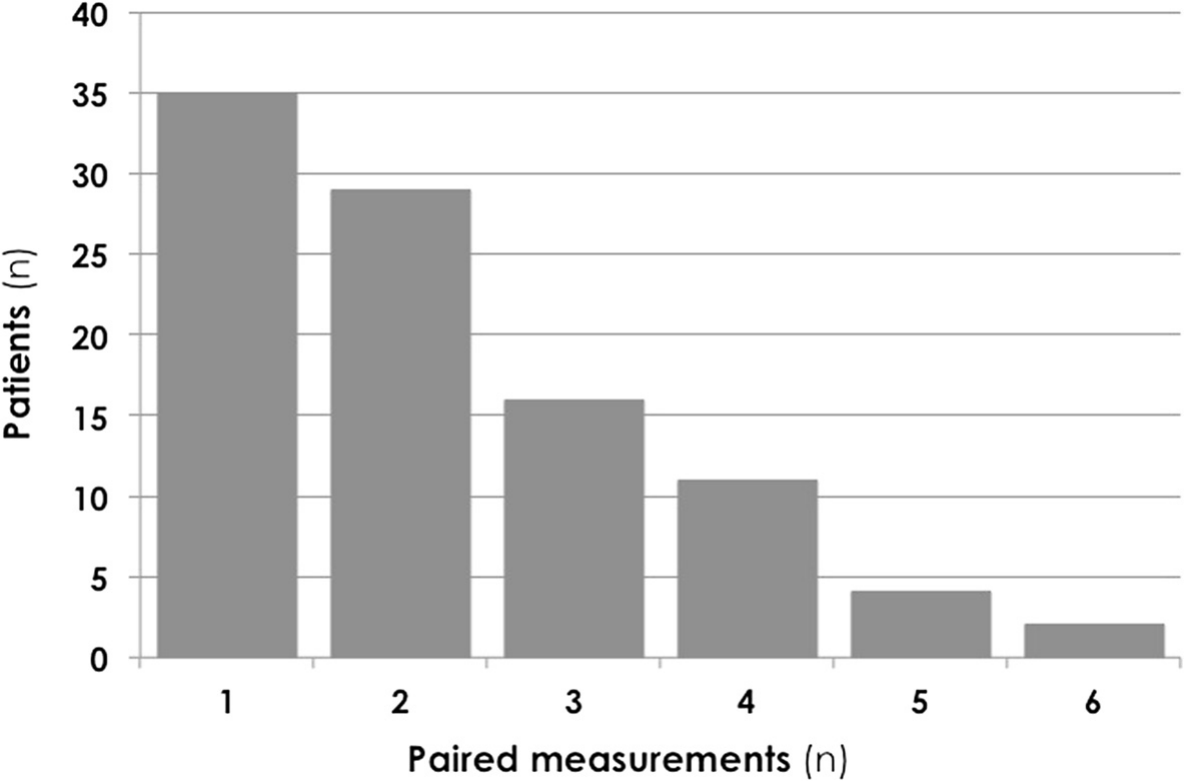
**Number of paired samples for each studied patient under the 24-hour follow-up period.** Each pair of measurements consists of two consecutive (3 ± 2 hours) simultaneous arterial and central venous blood samples, allowing for lactate clearance calculation.

During the follow-up period, 97 paired blood samples were obtained. The number of paired samples for each studied patient is shown in Figure 1. The elapsed time between consecutive measurements was 3 ± 2 hours. No improvement in lactate values was associated with higher P_cva_CO_2_/C_av_O_2_ ratio values in the previous measurement (1.9 ± 0.9 vs. 1.2 ± 0.4 mmHg · dl/ml O_2_, *P* <0.001), while P_cva_CO_2_ gap values did not statistically differ (6.0 ± 2.3 vs. 5.0 ± 2.1 mmHg, *P* = 0.08). Higher S_cv_O_2_ values were also observed in those patients whose lactate did not decrease within the following hours (73 ± 8% vs. 68 ± 9%, *P* = 0.01). When exploring the ability of these parameters in predicting adequate lactate clearance, for the whole 97 paired samples the P_cva_CO_2_/C_av_O_2_ ratio ROC analysis showed an area under the curve of 0.82 (95% confidence interval = 0.73 to 0.92, *P* <0.001), and a cutoff value of 1.4 had the best relationship between sensitivity (0.8) and specificity (0.75). The P_cva_CO_2_ gap ROC analysis showed an area under the curve of 0.62 (*P* = 0.07) for lactate improvement prediction. The odds ratio of an adequate lactate clearance was 0.10 (95% confidence interval = 0.03 to 0.3, *P* <0.001) in those patients with an elevated P_cva_CO_2_/C_av_O_2_ratio (≥1.4), whereas it did not reach statistical significance (*P* = 0.1) for those patients with a P_cva_CO_2_ gap ≥6 mmHg.

### Outcome

The mortality in the studied population was 29% (10 patients). Patients who died had similar lactate, S_cv_O_2_, and P_cva_CO_2_ gap values at inclusion, but showed significantly increased P_cva_CO_2_/C_av_O_2_ ratio values (1.9 ± 0.9 in nonsurvivors vs. 1.4 ± 0.45 in survivors, *P* = 0.03) (Table 2).

**Table 2:** **Patients’ main characteristics according to ICU survival**

	**Survivors ** **(** ***n*** **= 25)**	**Nonsurvivors ** **(** ***n*** **= 10)**	***P*** **value**
Age (years)	65 ± 13	67 ± 13	0.6
SAPS II	47 ± 10	53 ± 12	0.5
SOFA (day 1)	9 ± 3	9 ± 3	0.7
Heart rate (beats per minute)	103 ± 15	103 ± 12	0.9
MAP (mmHg)	81 ± 11	69 ± 12	0.07
Norepinephrine dose (mcg/kg/min)	0.85 ± 0.65	0.93 ± 0.73	0.7
Hemoglobin (g/dl)	11.7 ± 1.8	9.6 ± 2.1	0.1
S_cv_O_2_ (%)	71 ± 9	71 ± 6	0.9
Lactate (mg/dl)	25 ± 10	69 ± 83	0.8
P_cva_CO_2_ gap (mmHg)	5.4 ± 2.3	6.0 ± 1.5	0.3
P_cva_CO_2_/C_av_O_2_ ratio (mmHg · dl/ml O_2_)	1.4 ± 0.5	1.9 ± 0.9	0.03
ΔSOFA (day 4)	–3 ± 3	1 ± 4	0.02

Data presented as mean ± standard deviation or number (%). Main characteristics at inclusion are shown. During the follow-up period, the SOFA evolution within 4 days was also associated with higher mortality rates. MAP, mean arterial pressure; P_cva_CO_2_ gap, central venous-to-arterial carbon dioxide difference; P_cva_CO_2_/C_av_O_2_ ratio, central venous-to-arterial carbon dioxide difference/arterial-to-central venous oxygen content difference ratio; SAPS, Simplified Acute Physiological Score; ScvO_2_, central venous oxygen saturation; ΔSOFA, SOFA score at day 4 – SOFA score at day 1; SOFA, sequential Organ Failure Assessment.

## Discussion

The main result of our study is that, in a population of septic shock patients, once MAP and S_cv_O_2_ values were normalized, high P_cva_CO_2_/C_av_O_2_ ratio values were associated with the lack of lactate clearance within the following hours, and this condition was associated with patient mortality. In our population, the ability of the P_cva_CO_2_/C_av_O_2_ ratio to predict lactate evolution was stronger than that of the P_cva_CO_2_ gap. To our knowledge this is the first study exploring this issue, and our data validate that integration of the P_cva_CO_2_/C_av_O_2_ ratio within the resuscitation process of septic shock might have relevant clinical utility.

The present study aimed to add some information for a daily clinical question, such as whether to stop or continue resuscitating those patients suffering from septic shock once MAP and S_cv_O_2_ endpoints have been reached but lactate values are still elevated. According to current international guidelines [1], normalization of S_cv_O_2_ as a global marker for the adequacy of tissue oxygenation would be sufficient. On the other hand, using lactate clearance as guidance for the resuscitation process has been demonstrated to be as effective as S_cv_O_2_ guidance [3]. However, the real-time nature of S_cv_O_2_ monitoring has been decisive for its widespread use above lactate monitoring. In clinical practice, an adequate knowledge of the value and limitations of both variables would be desirable in order to understand each scenario, and take decisions accordingly. In recent years, some studies have corroborated the prognostic value of the P_cva_CO_2_ gap in several clinical conditions [11-14], and this parameter has been proposed as an additional marker of tissue perfusion adequacy [10]. Importantly, its value seems to persist even when S_cv_O_2_ has been normalized [11,13,14]. According to these observations, it has been suggested that adding the P_cva_CO_2_ gap as a supplementary endpoint in the resuscitation process of septic shock might prove beneficial [10], but to date prospective interventional studies exploring this issue are still lacking. Moreover, some authors have suggested that correcting the P_cva_CO_2_ gap by the C_av_O_2_ (P_cva_CO_2_/C_av_O_2_ ratio) might enhance its value as a marker of anaerobic metabolism [15,18]. The P_cva_CO_2_/C_av_O_2_ ratio has been proposed as an approximation of the respiratory quotient, the relationship between global carbon dioxide production and global oxygen consumption (VO_2_). According to Fick’s equation, VO_2_ is equal to the product of cardiac output and the C_av_O_2_. Similarly, global carbon dioxide production is equal to the product of cardiac output and the central venous-to-arterial carbon dioxide content difference. The respiratory quotient is therefore equal to the central venous-to-arterial carbon dioxide content difference/C_av_O_2_ ratio. Increased carbon dioxide production, relative to oxygen consumption, occurs under conditions of tissue hypoxia, and the presence of anaerobic metabolism may be inferred when the respiratory quotient rises above 1. Since over the physiological range of carbon dioxide contents the partial pressure of carbon dioxide is linearly related to carbon dioxide content, using the P_cva_CO_2_ as a surrogate for the carbon dioxide content difference has been previously accepted [15,18]. In the present study, we aimed at exploring whether an increased P_cva_CO_2_ gap and its combination with the C_av_O_2_ at the end of early goal-directed therapy would be predictive of an inadequate lactate clearance, and thus a ready-to-use tool for the decision-making process.

### The P_cva_CO_2_/C_av_O_2_ ratio as a marker of anaerobic metabolism

Mekontso-Dessap and coworkers suggested that the P_cva_CO_2_/C_av_O_2_ ratio might be a reflection of anaerobic metabolism, demonstrating a positive correlation between this parameter and lactate [15]. Indeed, in their study the P_cva_CO_2_/C_av_O_2_ ratio was superior to S_cv_O_2_ and the P_cva_CO_2_ gap for predicting elevated lactate values. Our study was designed to go one step further, and we explored the ability of the P_cva_CO_2_/C_av_O_2_ ratio to predict the adequacy of lactate clearance. Again, our results support the hypothesis that the P_cva_CO_2_/C_av_O_2_ ratio is a better predictor of anaerobic metabolism than the P_cva_CO_2_ gap. Of note, lactate values at inclusion were not associated with lactate evolution, strengthening the concept that an elevated lactate value at a given time point does not infer the presence of anaerobic metabolism. In a recent study, Monnet and coworkers nicely showed that, after performing a volume expansion, among those patients who were considered to be responders (increase in cardiac output) only patients with an elevated P_cva_CO_2_/C_av_O_2_ ratio at baseline increased their VO_2_ [18]. In other words, the ability to increase the metabolic rate, after increasing oxygen availability, was only observed in those patients with an altered P_cva_CO_2_/C_av_O_2_ ratio. According to their results, the authors introduced the idea that VO_2_ might only increase in response to an increase in global oxygen delivery when VO_2_ is limited (global oxygen delivery dependency), as suggested by an elevated P_cva_CO_2_/C_av_O_2_ ratio. Regrettably, we did not calculate VO_2_ or global oxygen delivery in our patients. However, on the whole our data are in accordance with these previous observations, and we might hypothesize that those patients whose P_cva_CO_2_/C_av_O_2_ ratio was high might have a limited VO_2_, causing anaerobic metabolism, and consequently were not able to decrease their lactate values within the following hours.

### Outcome

Although the present study was not powered to explore the prognostic value of the P_cva_CO_2_ gap and the P_cva_CO_2_/C_av_O_2_ ratio in terms of organ failure evolution or survival, our data suggest a significant association between mortality and the presence of an elevated P_cva_CO_2_/C_av_O_2_ ratio at the end of early goal-directed therapy. The association between the P_cva_CO_2_ gap and outcome has been demonstrated consistently in several scenarios, even in the presence of a normalized S_cv_O_2_ [11-14]. Although we were not able to reproduce the prognostic value of the P_cva_CO_2_ gap, our data suggest that the prognostic significance is enhanced when correcting the P_cva_CO_2_ gap for the oxygen content difference. Independently of our results regarding mortality, the demonstration that the P_cva_CO_2_/C_av_O_2_ ratio can predict the evolution of lactate strengthens its value, and brings this parameter closer to clinical practice. Since lactate clearance adequacy has already, and repeatedly, been associated with outcome [4,17], the ability to predict lactate evolution might prove useful in order to perform further resuscitation maneuvers, or to avoid unnecessary interventions and in doing so elude their potential deleterious effects.

### Study limitations

Several limitations might be taken into account when considering our results. Firstly, this is a single-center study, so our results might have limitations when trying to generalize for other ICUs or other settings. On the other hand, the homogeneity of our resuscitation process would strengthen the value of the observed results. Secondly, we failed to demonstrate a relationship between the P_cva_CO_2_ gap and lactate clearance, but the observed tendency to higher P_cva_CO_2_ gap values in those patients unable to decrease their lactate might suggest that the limited number of patients studied would account for this lack of significance. An additional limitation would derive from the lack of standardization of the time points of lactate measurement. Since this is an observational study, paired blood gas analyses were performed according to the medical team, without intervention from the researchers. Although current practice in our ICU includes the verification of a persistent normalized S_cv_O_2_ and confirming an adequate lactate clearance, the timing of this verification is inconstant. Therefore, our second pair of measurements was associated with time variability. Nevertheless, the elapsed time between measurements was 3 ± 2 hours, which is in accordance with previous clinical works prospectively evaluating lactate clearance and outcome [3,4]. When exploring the prognostic value of the P_cva_CO_2_/C_av_O_2_ ratio, we might be cautious with the observed results. As discussed above, the present study was not designed for this purpose, and the reduced sample size might limit the significance of the association with mortality, as suggested by the absence of prediction of mortality by the Simplified Acute Physiology Score II. Although the existing literature has proposed the P_cva_CO_2_/C_av_O_2_ ratio as a surrogate of the respiratory quotient, several situations might affect the relationship between carbon dioxide content and partial pressure of carbon dioxide, such as the Haldane effect [19]. Oxygen saturations were calculated from partial pressure of oxygen values, not measured by co-oximetry, with a potential source of error in the displayed ScvO_2_ values. Finally, we analyzed the value of the P_cva_CO_2_ gap and the P_cva_CO_2_/C_av_O_2_ ratio once the recommended S_cv_O_2_ goal was reached. However, since the S_cv_O_2_ value might be limited in septic conditions, when oxygen extraction deficit occurs, whether the P_cva_CO_2_ gap and the P_cva_CO_2_/C_av_O_2_ ratio might be useful independently of S_cv_O_2_ deserves further study. Regrettably, this issue was not addressed in our study.

## Conclusions

In a population of septic shock patients with normalized MAP and S_cv_O_2_, the presence of elevated P_cva_CO_2_/C_av_O_2_ ratio values significantly reduced the odds of adequate lactate clearance during the following hours. Integrating this parameter in future resuscitation algorithms might prove useful in order to obtain real-time information on the adequacy of tissue perfusion, helping in the decision-making process, such as when to continue resuscitating the tissue and/or when to stop interventions, despite high lactate levels.

## Key messages

- In the presence of an elevated P_cva_CO_2_/C_av_O_2_ ratio, the odds of adequate lactate clearance are significantly reduced.
- The P_cva_CO_2_/C_av_O_2_ratio might be a useful parameter in the resuscitation process, complimentary to ScvO_2_ and lactate.

## Abbreviations

C_av_O_2_: arterial-to-venous oxygen content difference
MAP: mean arterial pressure
P_cva_CO_2_ gap: central venous-to-arterial carbon dioxide difference
P_cva_CO_2_/C_av_O_2_: central venous-to-arterial carbon dioxide difference/arterial-to-venous oxygen content difference ratio
ROC: receiver operator characteristic
S_cv_O_2_: central venous oxygen saturation
VO_2_: global oxygen consumption

## References

[1] Dellinger RP, Levy MM, Rhodes A, Annane D, Gerlach H, Opal SM (2013). The Surviving Sepsis Campaign Guidelines Committee including The Pediatric Subgroup. Surviving Sepsis Campaign: International Guidelines for Management of Severe Sepsis and Septic Shock, 2012. Intensive Care Med.

[2] Mesquida J, Borrat X, Lorente JA, Masip J, Baigorri F (2011). Objectives of hemodynamic resuscitation. Med Intensiva..

[3] Jones AE, Shapiro NI, Trzeciak S, Trzeciak S, Arnold RC, Claremont HA (2010). Lactate clearance vs central venous oxygen saturation as goals of early sepsis therapy: a randomized clinical trial. JAMA..

[4] Jansen TC, van Bommel J, Schoonderbeek FJ, SleeswijkVisser SJ, van der Klooster JM, Lima AP (2010). Early lactate-guided therapy in intensive care unit patients: a multicenter, open-label, randomized controlled trial. Am J Respir Crit Care Med..

[5] Pope JV, Jones AE, Gaieski DF, Arnold RC, Trzeciak S, Shapiro NI (2010). Multi-center study of central venous oxygen saturation (ScvO2) as a predictor of mortality in patients with sepsis. Ann Emerg Med..

[6] Textoris J, Fouché L, Wiramus S, Antonini F, Tho S, Martin C (2011). High central venous oxygen saturation in the latter stages of septic shock is associated with increased mortality. Crit Care..

[7] van Beest P, Wietasch G, Scheeren T, Spronk P, Kuiper M (2011). Clinical review: use of venous oxygen saturations as a goal – a yet unfinished puzzle. Crit Care..

[8] Andersen LW, Mackenhauer J, Roberts JC, Berg KM, Cocchi MN, Donnino MW (2013). Etiology and therapeutic approach to elevated lactate. Mayo Clin Proc..

[9] Boyd JH, Forbes J, Nakada TA, Walley KR, Russell JA (2011). Fluid resuscitation in septic shock: a positive fluid balance and elevated central venous pressure are associated with increased mortality. Crit Care Med..

[10] Vallet B, Pinsky MR, Cecconi M (2013). Resuscitation of patients with septic shock: please ‘mind the gap’!. Intensive Care Med..

[11] Vallée F, Vallet B, Mathe O, Parraguette J, Mari A, Silva S (2008). Central venous-to-arterial carbon dioxide difference: an additional target for goal-directed therapy in septic shock?. Intensive Care Med..

[12] Futier E, Robin E, Jabaudon M, Guerin R, Petit A, Bazin JE (2010). Central venous O2 saturation and venous-to-arterial CO2 difference as complementary tools for goal-directed therapy during high-risk surgery. Crit Care..

[13] van Beest PA, Lont MC, Holman ND, Loef B, Kuiper MA, Boerma EC (2013). Central venous-arterial pCO2 difference as a tool in resuscitation of septic patients. Intensive Care Med..

[14] Ospina-Tascon GA, Bautista-Rincon DF, Umaña M, Tafur JD, Gutierrez A, Garcia AF (2013). Persistently high venous-to-arterial carbon dioxide differences during early resuscitation are associated with poor outcomes in septic shock. Crit Care..

[15] Mekontso-Dessap A, Castelain V, Anguel N, Bahloul M, Schauvliege F, Richard C (2002). Combination of venoarterial PCO2 difference with arteriovenous O2 content difference to detect anaerobic metabolism in patients. Intensive Care Med..

[16] Levy MM, Fink MP, Marshall JC, Abraham E, Angus D, Cook D (2003). 2001 SCCM/ESICM/ACCP/ATS/SIS International Sepsis Definitions Conference. Crit Care Med..

[17] Nguyen HB, Rivers EP, Knoblich BP, Jacobsen G, Muzzin A, Ressler JA (2004). Early lactate clearance is associated with improved outcome in severe sepsis and septic shock. Crit Care Med..

[18] Monnet X, Julien F, Ait-Hamou N, Lequoy M, Gosset C, Jozwiak M (2013). Lactate and venoarterial carbon dioxide difference/arterial-venous oxygen difference ratio, but not central venous oxygen saturation, predict increase in oxygen consumption in fluid responders. Crit Care Med..

[19] Jakob SM, Kosonen P, Ruokonen E, Parviainen I, Takala J (1999). The Haldane effect – an alternative explanation for increasing gastric mucosal PCO2 gradients?. Br J Anaesth..

